# Stricture of the Rectum

**Published:** 1856-10

**Authors:** Alanson Abbe

**Affiliations:** Boston


*Stricture of the Rectum.* By Alanson Abbe, M. D., Boston.

Mrs. C. R. D., aged 24, the mother of one child, three years of age, has for many years been afflicted with obstinate and unyielding costiveness, attended with hæmorrhoids unusually painful and distressing. Her bowels were evacuated, usually, once in eight or ten days, and only by the aid of cathartics. Her general health was much impaired; there were daily paroxysms of severe pain in the head, giddiness, pain in the loins and anus; she was excessively irritable, though her appetite and digestive organs were uniformly good.

April, 1856.—I was called to see her. On examination, I found five large hæmorrhoids on the verge of the anus, from which, apparently, issued a constant discharge of pus. The hæmorrhoids were removed by ligature, with no unusual symptoms, and healed kindly and well, yet the discharge continued undiminished. On examination per rectum, I discovered a stricture of the rectum, about two and a half inches from the sphincter ani, the diameter of which, under force, did not exceed one-fourth of an inch. Pus oozed constantly through this orifice. I was wholly unable to ascertain the condition of the intestine above the stricture. On reflection, I concluded to dilate the stricture, instead of dividing or cauterizing it, and for this purpose obtained of Dr. Codman seven conical tents, made of the bark of slippery elm, as recommended by Dr. Storer.

May 5th.—Introduced an elm tent into the stricture, letting it rest on the inner surface of the sphincter ani, and secured it by a T bandage, a compress of cotton resting upon the anus.

May 6th.—Early in the morning the tent was discharged, with a small portion of fæcal matter. At 11, A. M., another tent of larger size was introduced in the same manner, and secured as before.

7th.—The tent came away this morning, followed by a moderate discharge of fæces. I introduced another tent of still larger size, and secured it in a like manner as the others.

8th.—The discharge of the tent passed this morning as the others had done, with very little fæcal matter, but a large discharge of pus. Directed an enema of mucilege gum acacia, and the comp. inf. senna, ℥ iv.

9th.—On examination of the condition of the stricture, found it enlarged sufficiently to admit my fore finger, with some force, to the first joint. Introduced the fourth tent, the largest diameter of which was about an inch. While introducing it, felt the stricture suddenly give way, presenting no further resistance. This instant yielding of the stricture produced a temporary faintness of the patient, which passed off in three or four minutes.

10th.—Very early this morning, the disposition to use the stool became so urgent, that she was compelled to remove the bandage and let the tent pass, and with it came a free and copious discharge of fæces and pus. On examination, found the stricture nearly overcome. Above it, as far as the finger could reach, the intestine was indurated, granulated and ulcerated. The discharge of pus was evidently from this source, occasioned by retention of fæces above the stricture.

19th.—Patient reports herself well; has a free and easy discharge from her bowels, daily. She discovers no signs of purulent matter in her evacuations, or on her linen.—*Boston Med. and Surg. Journ.*

